# Estimating nonlinear anisotropic properties of healthy and aneurysm ascending aortas using magnetic resonance imaging

**DOI:** 10.1007/s10237-024-01907-6

**Published:** 2024-11-26

**Authors:** Álvaro T. Latorre Molins, Andrea Guala, Lydia Dux-Santoy, Gisela Teixidó-Turà, José Fernando Rodríguez-Palomares, Miguel Ángel Martínez Barca, Estefanía Peña Baquedano

**Affiliations:** 1 Aragón Institute for Engineering Research (I3A), University of Zaragoza, Zaragoza, Spain; 2 Vall d’Hebron Institut de Recerca, Barcelona, Spain; 3 Biomedical Research Networking Center on Cardiovascular Diseases (CIBER-CV), Instituto de Salud Carlos III, Madrid, Spain; 4 Department of Cardiology, Hospital Universitari Vall d’Hebron, Barcelona, Spain; 5 Departament de Medicina, Universitat Autónoma de Barcelona. Bellaterra, Barcelona, Spain; 6 Biomedical Research Networking Center in Bioengineering, Biomaterials and Nanomedicine (CIBER-BBN), Zaragoza, Spain

**Keywords:** Ascending aorta, Inverse modeling, Mechanical characterization, Nonlinear

## Abstract

An ascending aortic aneurysm is an often asymptomatic localized dilatation of the aorta. Aortic rupture is a life-threatening event that occurs when the stress on the aortic wall exceeds its mechanical strength. Therefore, patient-specific finite element models could play an important role in estimating the risk of rupture. This requires not only the geometry of the aorta but also the nonlinear anisotropic properties of the tissue. In this study, we presented a methodology to estimate the mechanical properties of the aorta from magnetic resonance imaging (MRI). As a theoretical framework, we used finite element models to which we added noise to simulate clinical data from real patient geometry and different properties of healthy and aneurysmal aortic tissues collected from the literature. The proposed methodology considered the nonlinear properties, the zero pressure geometry, the heart motion, and the external tissue support. In addition, we analyzed the aorta as a homogeneous material and as a heterogeneous model with different properties for the ascending and descending parts. The methodology was also applied to pre-surgical,*in vivo* MRI data of a patient who underwent surgery during which an aortic wall sample was obtained. The results were compared with those obtained from *ex vivo* biaxial test of the patient’s tissue sample. The methodology showed promising results after successfully recovering the nonlinear anisotropic material properties of all analyzed cases. This study demonstrates that the variable used during the optimization process can affect the result. In particular, variables such as principal strains were found to obtain more realistic materials than the displacement field.

## Introduction

Ascending thoracic aortic aneurysm (ATAA) is a frequently asymptomatic condition characterized by the localized dilatation of the aorta. People with hypertension or inherited connective tissue disorder, such as Marfan or Ehlers-Danlos syndromes, are more likely to develop ATAA (Pape et al. 2007). Although the incidence of this condition is only 5.3 occurrences per 100,000 individuals per year (Melo et al. 2022), the rupture of the ATAA results in death in the vast majority of the cases (Bickerstaff et al. 1982; Johansson et al. 1995; Melo et al. 2022). Nevertheless, the prognosis for patients with ATAA improves if they receive treatment before complications arise (Clouse et al. 1998). Therefore, a proper follow-up of patients diagnosed with ATAA could control the risk of rupture or dissection (Sharples et al. 2022). In clinical practice, patients are considered for surgical intervention when the ATAA maximum diameter reaches the critical size of 55 mm (Saliba et al. 2015). However, it was reported that aortic aneurysms with lower diameters could rupture, and some studies showed the diameter has limited performance to predict the risk of rupture (Pape et al. 2007; Elefteriades and Farkas 2010). Moreover, this measurement is affected by the resolution of the imaging technique, measurement conventions, and cardiac cycle phase and is subject to inter-observer variability (Elefteriades and Farkas 2010; Dux-Santoy et al. 2022). Consequently, new guidelines should consider further patient characteristics (Sharples et al. 2022), and mechanical properties of the aorta could become a useful intervention criterion (Elefteriades and Farkas 2010). 

Mechanically, the ATAA ruptures when the stress to which the aortic wall is subjected exceeds its mechanical strength. For abdominal aortic aneurysms, studies reported that peak wall stress is a more reliable risk criterion than measured diameter (Fillinger et al. 2003; Vande Geest et al. 2006). That is why patient-specific finite element (FE) models could play a crucial role in estimating the stress distribution across the aortic wall, facilitating the estimation of rupture risk (Trabelsi et al. 2016) or the rupture location (O’Rourke et al. 2022). Nonetheless, peak wall stress alone is insufficient as a criterion for estimating the vulnerability, as it must be correlated with the patient’s specific wall strength (Smoljkic et al. 2017). Some studies noted that local stiffness exhibits a correlation with rupture criterion (Duprey et al. 2016; Trabelsi et al. 2018; Farzaneh et al. 2019b). Therefore, to obtain a good estimation of the risk of rupture, it is necessary to know the arterial wall material and its stress state. Inverse analysis methods are widely used to recover material properties from medical imaging (Auricchio et al. 2015; Trabelsi et al. 2016; Liu et al. 2017, 2019; Farzaneh et al. 2019b; Laita et al. 2024). Those methods usually need the acquisition of two clinical images, normally in systolic and diastolic pressures (Trabelsi et al. 2016; Liu et al. 2017; Farzaneh et al. 2019b). Then, the relative displacements or superficial strains between the aortic wall at those pressures are computed by image registration (Liu et al. 2019; Zhang 2021). The next step consists of estimating the mechanical properties through an optimization process, where the measured displacements/strains are compared with those computed by an inverse finite element analysis (Farzaneh et al. 2019b; Liu et al. 2019; Torun et al. 2022). Clinical imaging, such as computed tomography (CT) or magnetic resonance imaging (MRI), visualizes *in vivo* images of the aorta, delivering pressurized instead of unpressurized geometries. Some approaches used pressurized images to estimate the linear elasticity of the tissues (Auricchio et al. 2015; Farzaneh et al. 2019b). However, this approximation could lead to an overestimation of the relative stiffness (Latorre et al. 2023). In order to consider the nonlinear properties of the material, it is necessary to start from an undeformed configuration. Therefore, it is common to employ a Pull-Back algorithm to estimate the unpressurized geometry (Raghavan et al. 2006; Riveros et al. 2013; Bols et al. 2013). Moreover, using patient-specific, nonlinear material properties have been proven to have the greatest impact on the peak wall stress (Trabelsi et al. 2015). Trabelsi et al. (2016), developed an *in vivo*, non-invasive method to obtain the patient-specific material properties of the ATAA from CT. However, they assumed a hyperelastic isotropic behavior of the wall, while the collagen orientation is relevant and plays a key role in the mechanical behavior of the ascending aorta (Cosentino et al. 2023). Due to the high computational cost of inverse modeling, Liu et al. (2019) used a multi-resolution direct search that enabled them to quantify the Gasser-Ogden-Holzapfel (GOH) material parameters (Gasser et al. 2006) from *in vivo* aortic CT in 1$\sim$2 h. However, it was reported the importance of considering heterogeneous properties, rather than assuming homogeneous properties throughout the aortic geometry (Farzaneh et al. 2019a, b). The majority of these approaches consider a homogeneous behavior of the aorta (Liu et al. 2019; Zhang 2021), and those that consider different properties were linear elastic or isotropic behavior (Farzaneh et al. 2019a; Trabelsi et al. 2016). Other approaches obtained the nonlinear anisotropic material parameters of the GOH strain density function with CT and using pressure-diameter curves and intraoperative measurement of the wall thickness (Smoljkic et al. 2017) or used machine learning techniques to estimate stress levels and the area of rupture (He et al. 2021). Nevertheless, these studies focus on specific regions of the aorta, which made it possible to visualize the tension only at a localized level.

In previous work, our group developed a methodology to estimate the nonlinear properties of atherosclerotic tissues from intravascular ultrasound images (IVUS) (Latorre et al. 2023). The technique was developed for 2D data and considering isotropic tissue behavior due to the nature of the pathology. In this paper, we present a theoretical methodology to obtain the nonlinear anisotropic properties of the ATAA from 3D MRI. After segmenting the aorta at the diastolic and systolic phases, its relative displacements were estimated through non-rigid registration. Then, through an optimization process, we recovered the GOH parameters by minimizing the error between the measured relative displacements or strains and those computed by inverse FE modeling. We studied the influence of using displacement or different deformation fields as variables to minimize the optimization process. On each iteration, we analyze a different set of material parameters, taking into consideration external tissue support, heart movement, and the unpressurized geometry. First, as a theoretical framework, we worked with FE models to simulate MRI data of ATAA using real geometry and materials collected from the literature. We analyzed aortas assuming homogeneous and heterogeneous materials. In the first case, we impose a single healthy or diseased material for the whole aorta, while in the second we differentiate between ascending and descending aorta materials. Then, we obtained the nonlinear material properties and their respective zero-pressure geometry, which enabled us to estimate the stress distribution over the arterial wall and thus information on the risk of rupture. Finally, we validated the methodology by implementing it on a real patient who underwent surgical resection of the aneurysmatic ascending aorta. Therefore, we compared the *in vivo* results with those obtained from *ex vivo* biaxial testing.

## Material and methods

This section is divided into four main parts. First, we described the data acquisition and its preprocessing to obtain a segmentation of the aorta and the relative displacements between diastolic and systolic pressures. Secondly, we generated FE models with the diastolic geometry and imposed the physiological loads and boundary conditions. Then, we presented the inverse method to estimate the nonlinear anisotropic properties at the zero-pressure geometry. In the last section, we explained the *in vivo-ex vivo* application of the methodology.

### Data acquisition and preprocessing

**Fig. 1:**
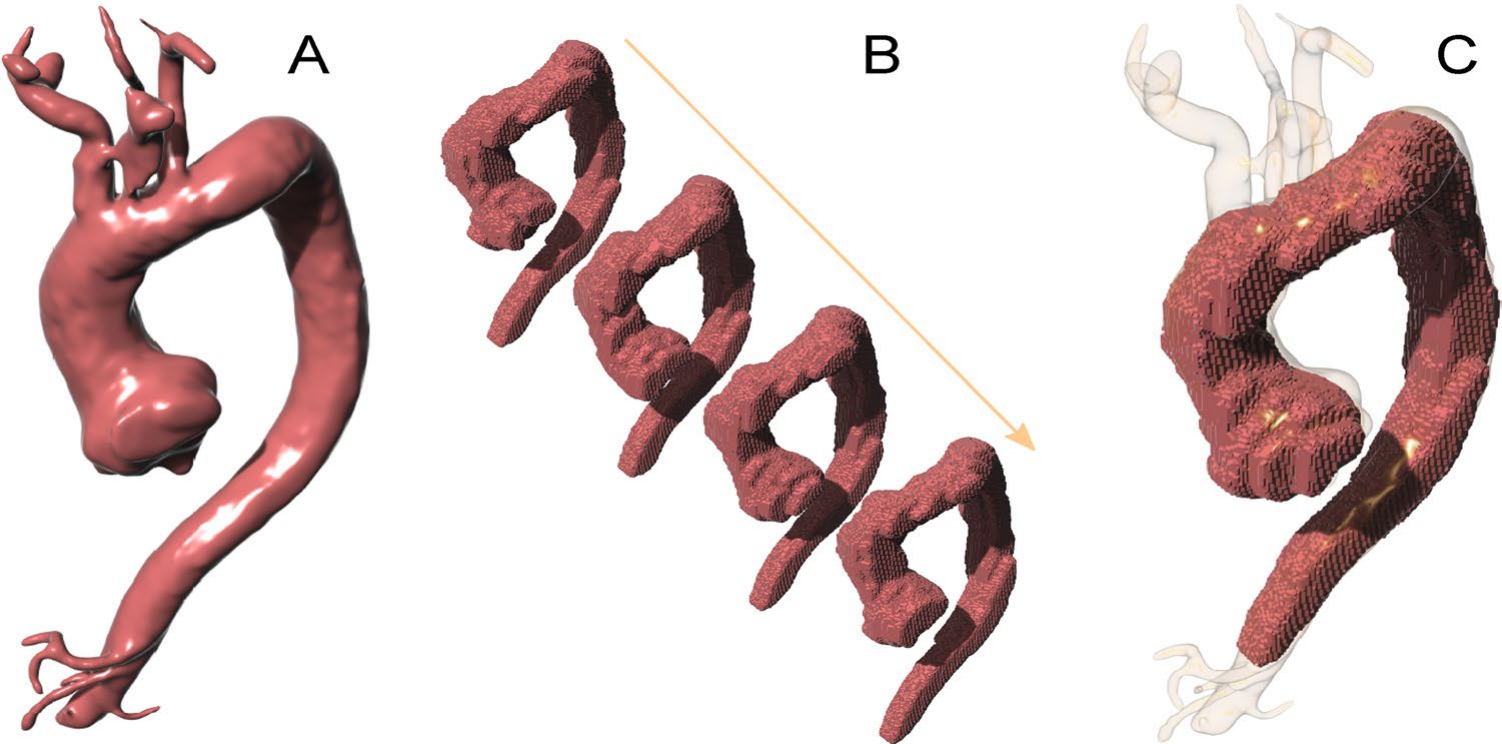
Segmentation of the Aorta: **A** from MRA; **B** from sagittal cine. **C** Sagittal cine segmentation correlating with the MRA segmentation

MRI data were derived from a patient who underwent surgical repair of ATAA at the Vall d’Hebron University Hospital (Barcelona, Spain). The study was approved by the local ethics committee (PR(AG)23/2018) and written informed consent was obtained from the patient. MRI was performed on a clinical 1.5 T scanner (Signa, GE Healthcare, Waukesha, Wisconsin, USA). The protocol included a stack of balanced steady-state free-precession (bSSFP) cine images (in-plane spatial resolution of 1.87 mm, through-plane spatial resolution of 8 mm, no gap, 30 phases) and a contrast-enhanced angiography (MRA, in-plane spatial resolution of 0.8 mm, through-plane spatial resolution of 3 mm, no gap) covering the whole thoracic aorta in sagittal view. We employed high-resolution MRA to create the geometry of our model, and the cine images to estimate the displacement fields. The aorta was segmented in both, the MRA and each image of the sagittal cine, using the open-access software MITK (Wolf et al. 2004). These segmentations were semi-automatically performed by using the threshold method. We also applied smooth treatments over the segmented surfaces using Rhinoceros 3D (McNeel et al. 2010). Figure 1. A and B show the aortic segmentation in the MRA and sagittal cine, respectively. We used the segmentations of the sagittal cine to identify the systolic and diastolic geometries. The segmentation with the lowest volume was defined as the diastolic geometry at 80 mmHg, while the highest volume was considered as systolic geometry at 120 mmHg (Farzaneh et al. 2019a; Trabelsi et al. 2015). Then, we searched for the cine phase that most closely resembles the aorta as depicted by MRA (Fig. 1**C**). Relative displacements between systolic and diastolic phases ($u_{diast\rightarrow syst}$) and between diastole and MRA ($u_{diast\rightarrow mra}$) were estimated using a non-rigid ICP (Iterative Closest Point) registration algorithm (Audenaert et al. 2019; Audenaert 2023). This algorithm allowed us to obtain the transformation of a point cloud from an initial state (diastole) to a target state (systole). As the relative displacements were computed in the sagittal low-resolution segmented cine, we interpolated the displacement solution over the MRA segmented mesh. Finally, with those displacements, we were able to achieve a high-resolution geometry at diastolic pressure ($\Omega _{diast}$) and the relative displacement between diastole and systole.

After smoothing the end-diastolic geometry in Rhinoceros 3D, we trimmed the region proximal to the aortic root, the arteries branching in the aortic arch, and the descending aorta distal to the diaphragm. Although we focused on the ascending part of the geometry, we also maintained the descending part to analyze areas with different materials. As the segmentation performed over the MRA gave us the lumen geometry, we added a uniform thickness of 2 mm to consider the aortic wall (O’Rourke et al. 2022). Finally, we meshed the geometry with a tetrahedral mesh using the open-access software NetGen. The mesh had a total of 5600 nodes and the element type chosen was the hybrid linear tetrahedral elements (C3D4H). The mesh size was adjusted to have a balance between the approximation of the results and the computational cost during the inverse analysis.

### FE modeling

This methodology aimed to directly obtain the material parameters that describe the mechanical properties from MRI data. However, as a preliminary theoretical framework to develop the methodology, we worked with FE models to generate simulated MRI data of ATAA using a real patient-specific geometry, and materials collected from the literature (Haskett et al. 2010; Weisbecker et al. 2012; Smoljkic et al. 2017; Mousavi and Avril 2017). First, we imported the mesh of the diastolic aorta into the software Abaqus CAE 6.14 (*Dassault Systems 2014*). The aorta presents a nonlinear anisotropic behavior (Gasser 2017), so we used the GOH strain energy density function implemented in Abaqus (Eq. 1) (Gasser et al. 2006). This equation has three terms. First, the volumetric term, where the parameter *D* stands for the compressibility of the material, in this case, it was fixed to zero to reproduce the incompressibility of the arterial tissue (Carew et al. 1968). Meanwhile, in the isotropic term, $C_{10}$ refers to the initial stiffness of the tissue. The anisotropic term is described by $k_{1}$ and $k_{2}$ that represent the stiffness at high pressures and the shape of the exponential curves, respectively. The parameter $\kappa$ represents the collagen fiber dispersion. Table 1 shows the GOH parameters obtained from the literature, where $\alpha$ is the preferred collagen orientation to the circumferential direction. We considered two different scenarios: homogeneous models with ascending aorta properties, both healthy and diseased; and heterogeneous cases with healthy and diseased ascending and healthy descending thoracic aorta properties.

1 $$\begin{aligned} \Psi = & \frac{1}{D} \cdot \left[ {J - 1} \right]^{2} + C_{{10}} \left[ {I_{1} - 3} \right] \\ & + \frac{{k_{1} }}{{2k_{2} }}\sum\limits_{{i = 4,6}} {\left( {\exp \left( {k_{2} \left[ {\kappa \left[ {I_{1} - 3} \right] + \left[ {1 - 3\kappa } \right]\left[ {I_{i} - 1} \right]} \right]^{2} } \right) - 1} \right)} , \\ \end{aligned}$$

**Table 1:** GOH material parameters obtained from the literature. All samples were human samples and were tested *ex vivo*. AA: Ascending Aorta, TA: Thoracic Aorta, ATAA: Ascending Thoracic Aorta Aneurysm, DTA: Descending Thoracic Aorta

${\textbf {n}}^{\underline{\textrm{O}}}$	$C_{10}$ **[kPa]**	$k_{1}$ **[kPa]**	$k_{2}$ **[-]**	$\kappa$ **[-]**	$\alpha [^{\circ }]$	**Comments**	**References**
1	20.9	710	0.4	0.08	44.4	Healthy AA. 0–30 years	Haskett et al. (2010)
2	16.6	790	3.13	0.13	44.1	Healthy AA. 31–60 years	Haskett et al. (2010)
3	44.6	2070	93.33	0.18	45.5	Healthy AA. Over 61 years	Haskett et al. (2010)
4	16.05	2035.9	14.16	0.33	90	ATAA	Smoljkic et al. (2017)
5	10.9	477.6	20.19	0.31	90	ATAA	Smoljkic et al. (2017)
6	7.5	910	71.8	0.32	38.6	TA	Weisbecker et al. (2012)
7	16.5	1030	36.3	0.08	48.5	TA	Weisbecker et al. (2012)
8	7.5	200	6.92	0.28	53.9	TA	Weisbecker et al. (2012)
9	4.65	293	0.1	0	45	ATAA	Mousavi and Avril (2017)
10	30	500	5	0	35	–	Farzaneh et al.( 2019a)
11	29.7	1340	1.79	0.1	44.5	Healthy DTA. 0–30 years	Haskett et al.( 2010)
12	35.1	1930	39.64	0.2	32.9	Healthy DTA. 31–60 years	Haskett et al. (2010)
13	63.3	3220	145.35	0.2	55.2	Healthy DTA. Over 61 years	Haskett et al. (2010)

In order to mimic the physiologic conditions of the aorta, we took into account the external tissue support such as the spine contact. The majority of the approaches in literature neglect this tissue support, which could lead to an artificial motion pattern (Moireau et al. 2012). We implemented non-uniform Robin conditions through the domain, differentiating three areas: contact with the spine, near the spine, and the remaining tissue (Moireau et al. 2012; Pagoulatou et al. 2021). We imposed an external elastic foundation with different stiffness: $k=10000\,\mathrm{{mN/mm}}$ for the spine contact, $k=10\,\mathrm{{mN/mm}}$ near the spine, $k=0.1\,\mathrm{{mN/mm}}$ for the remaining tissue. These coefficients were defined by Moireau et al. (2012) as the average values and the location of the different external fixation was chosen by analyzing the MRI data. For instance, spine contact was imposed on the external surface with less displacement measured in the MRI data with the ICP algorithm. To recover the unpressurized geometry from the segmented diastolic state, we introduced a Pull-Back algorithm. This step was based on the fixed point algorithm defined by Raghavan et al. (2006), and allowed us to get the zero-pressure (ZP) geometry through an iterative process. In this process, we added 80 mmHg to the diastolic geometry, $\Omega _{\mathrm{{diast}}}$, and computed the displacements ($u_{\mathrm{{diast}}\rightarrow 80\,mmHg}$). Then, we obtained an estimation of the ZP geometry ($\Omega _{zp}$) by imposing the displacements to the diastolic configuration multiplied by the recovery factor ($k_{zp}$), as can be appreciated in Eq. 2. The final ZP configuration was achieved through an iterative process by varying the recovery factor and comparing the error of the coordinates between the ZP geometry candidate, after adding 80 mmHg, and the diastolic geometry. Equation 3 shows the calculation of the error between the target coordinates ($\Omega _{\mathrm{{diast}}}$) and the evaluated coordinated of each iteration ($\Omega _{\mathrm{{zp+80mmHg}}}$), where *N* represents the number of nodes in the FE models. The mean error obtained in the different models was $1.36 \pm 0.21\%$.

2 $$\Omega _{{{\text{zp}}}} = \Omega _{{{\text{diast}}}} - k_{{{\text{zp}}}} \cdot u_{{{\text{diast}} \to 80{\kern 1pt} {\text{mmHg}}}} {\text{ }}$$

3 $${\text{Error}}_{{{\text{zp}}}} = 100 \cdot \sqrt {\frac{{\sum\limits_{{i = 1}}^{N} | \Omega _{{{\text{diast}}}} (i) - (\Omega _{{{\text{zp}} + 80{\text{mmHg}}}} (i))|^{2} }}{N}} \cdot \frac{1}{{{\text{mean}}(\Omega _{{{\text{diast}}}} )}}{\text{ }}$$

**Fig. 2:**
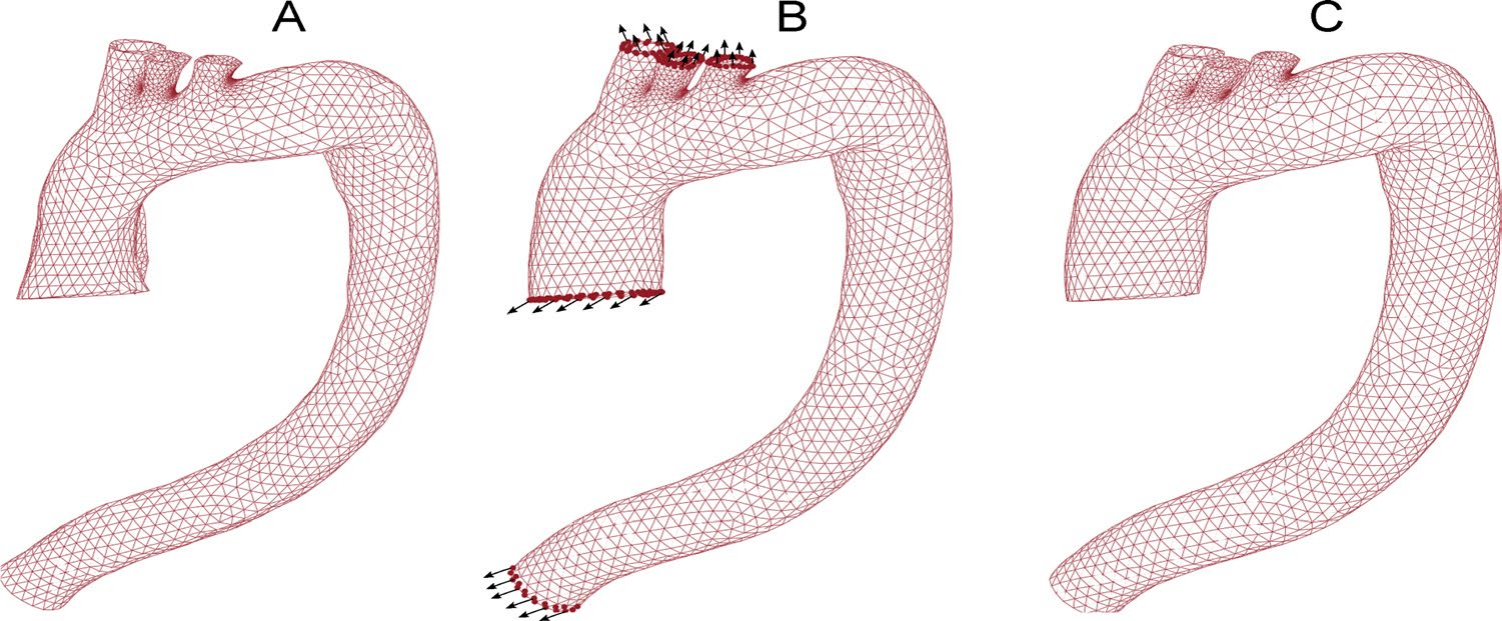
**A** Tetrahedral mesh of the unpressurized geometry. **B** Diastolic geometry after the first step, where the dots represent nodes used to impose displacements measured in the MRI as a boundary condition. **C** Systolic geometry obtained after the second step

Then, a FE model was built to get the systolic state from the ZP geometry. The inflation of the aorta was divided into two steps. Firstly, we simulated aortic inflation from the unpressurized configuration to the diastolic state. For this purpose, we added an internal pressure of 80 mmHg and fixed the node displacements of the ascending and descending ends and branches of the aortic arch. Figure 2.(A) and (B) show the unpressurized and diastolic geometries. Once the diastolic state was obtained, we introduced the remaining 40 mmHg of pressure to reach a systolic pressure of 120 mmHg. In addition to the load, the measured displacements from the sagittal cine, $u_{diast\rightarrow syst}$, were enforced as boundary conditions over the proximal aortic nodes to consider the heart movements (Pagoulatou et al. 2021). We also imposed the measured displacements of the descending and branch nodes to consider physiological displacements resulting from regions beyond the analysis domain. Figure 2.(B) illustrates the diastolic configuration, with nodes where displacements $u_{\mathrm{{diast}}\rightarrow syst}$ were applied, marked as dots. Both steps had the external tissue support defined by Robin conditions. Finally, at the end of the simulation, we obtained the systolic configuration represented in Fig. 2.(C). These FE results were used only to simulate the data derived from MRI. Therefore, only the FE relative displacements between diastolic-systolic states ($u_{\mathrm{{diast}}\rightarrow syst}^{'}$) were kept. Lastly, since the estimated displacement obtained from the ICP algorithm had some background noise, we added 20 dB of signal-to-noise ratio in the displacement field (Porée et al. 2015; Latorre et al. 2023). We referred to this displacement field as ground truth FE displacements and used it in the theoretical development of the methodology as if it were the displacement obtained from MRI.

### Mechanical characterization

In this section, we detail how we implemented an inverse model to estimate the nonlinear anisotropic properties of the aortic material using MRI data. In previous work, our group developed an optimization process to recover the nonlinear isotropic GOH parameters of atherosclerotic tissues in coronary arteries from 2D IVUS images (Latorre et al. 2023). This process was updated for 3D aortic geometries. Furthermore, atherosclerotic tissues were considered as having an isotropic behavior by fixing the fiber dispersion, $\kappa$, to 0.333 with no relevance for the angle orientation $\alpha$. Conversely, the mechanical response of aortic tissues is highly dependent on the fiber orientation. The present optimization was performed by linking Matlab and Abaqus software. In the process, we tried to match a variable obtained by the FEM during iteration with the same variable derived from MRI data. As explained in the previous section, the displacements between systole and diastole can be estimated from clinical data through registration techniques. In this article, we employed the real displacement field to impose the boundary conditions of our model, and we used FE models to simulate the MRI-displacements with different tissues. Therefore, the displacement field was used as the target for the optimization process trying to match the iterated displacements ($u_{diast\rightarrow syst}^{iter}$) with the ground truth FE displacements ($u_{diast\rightarrow syst}^{mri'}$). In addition, in the *in vivo* case, we used the displacements registered in the MRI as a target of the optimization process. Another variable occasionally employed in previous works for the optimizations is the strain. This mechanical variable has been successfully used in other studies with the advantage of remaining unaffected by the rigid movement (Zhang 2021). Strain fields could be obtained from the measured displacement field. Equation 4 shows how to compute the deformation gradient tensor, ***F***, using the diastolic configuration and the relative displacements between diastolic and systolic phases. Then, Eq. 5 shows how to compute the logarithmic strains tensor, ***LE***, from ***F***, which are a common variable used in large deformation problems in engineering. We obtained the maximum logarithmic strains ($LE_{max}$) and the logarithmic strains at the midpoint of the element ($LE_{mid}$). These values possess the advantage of being invariants, so their values did not depend on the reference coordinate system of the MRI data. Therefore, we proposed three possible variables to minimize in the optimization process, the modulus of the relative displacements ($u_{diast\rightarrow syst}$), and the max and mid logarithmic strains. First, we used synthetic data as the theoretical framework, employing FE strains as the ground truth ($LE_{max}^{{mri}'}$ and $LE_{mid}^{{mri}'}$). We defined our cost function, $J_{0}$, as the normalized root mean squared error between the measured and the iterated displacement and strain variable, as seen in Eq. 6,7 and 8 where *N* refers to the number of nodes in the FE model. As MRI only provided information about the lumen and not the aortic wall, we only analyzed the displacements corresponding to the lumen nodes.

4 $${\mathbf{F}} = \frac{{\partial \Omega _{{{\text{syst}}}} }}{{\partial \Omega _{{{\text{diast}}}} }} = \frac{{\partial \left( {\Omega _{{{\text{diast}}}} + u_{{{\text{diast $\rightarrow$ syst}}}} } \right)}}{{\partial \Omega _{{{\text{diast}}}} }}{\text{ }}$$

5 $$\begin{aligned} & {\textbf {LE}}=\frac{1}{2}\cdot log\left({\textbf {F}}^{T}\cdot {\textbf {F}}\right) \end{aligned}$$

6 $$J_{0}^{u} \left( {u_{{{\text{diast}} \to {\text{syst}}}}^{{{\text{mri}}^{\prime } }} ,u_{{{\text{diast}} \to {\text{syst}}}}^{{{\text{iter}}}} } \right){\text{ }} = 100\frac{\sqrt {\frac{1}{N}\sum {\left( {u_{{{\text{diast}} \to {\text{syst}}}}^{{{\text{mri}}^{\prime } }} (i) - u_{{{\text{diast}} \to {\text{syst}}}}^{{{\text{iter}}}} (i) } \right)^{2}} }}{mean(u^{mri'}_{diast\rightarrow syst})}$$

7 $$J_{0}^{LE_{max}} \left( {LE_{{{\text{max}}}}^{{{\text{mri}}^{\prime } }} ,LE_{max}^{{{\text{iter}}}} } \right){\text{ }} = 100\frac{\sqrt {\frac{1}{N}\sum {\left( {LE_{max}^{{{\text{mri}}^{\prime } }} (i) - LE_{max}^{{{\text{iter}}}} (i) } \right)^{2}} }}{mean(LE^{mri'}_{max})} ,$$

8 $$J_{0}^{LE_{mid}} \left( {LE_{{{\text{mid}}}}^{{{\text{mri}}^{\prime } }} ,LE_{mid}^{{{\text{iter}}}} } \right){\text{ }} = 100\frac{\sqrt {\frac{1}{N}\sum {\left( {LE_{mid}^{{{\text{mri}}^{\prime } }} (i) - LE_{mid}^{{{\text{iter}}}} (i) } \right)^{2}} }}{mean(LE^{mri'}_{mid})} ,$$

**Fig. 3:**
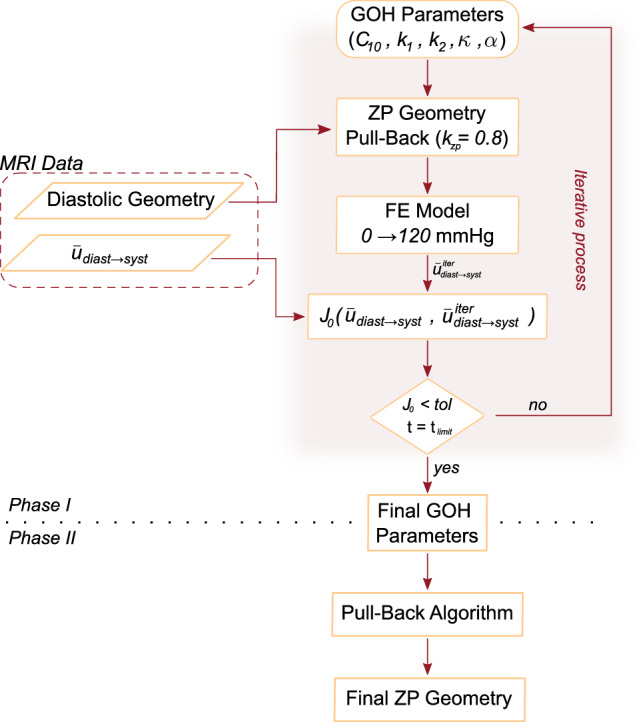
Scheme of the inverse modeling process

We employed a pattern-search algorithm, a direct search optimization method that divides the space of the cost function into mesh points (Hooke and Jeeves 1961). It is not based on gradients and works with smooth and non-smooth functions. It uses an initial point and starts evaluating different points depending on the selected polling method. We chose a generating set search poll method that reported good results (Latorre et al. 2023). Before the optimization process, a sensitivity analysis of the different GOH parameters was conducted to evaluate the relevance of each parameter in the cost function. The most relevant variables (shown in the Results section) were given a higher weight during the optimization. The algorithm also needed a search range for each GOH parameter. The ranges were $C_{10}$ [10–240] kPa, $k_{1}$ [5–8000] kPa, $k_{2}$ [1–240], $\kappa$ [0$-$0.333], and $\alpha$ [0–90]°. The iterative process ended when the tolerance in the cost function was less than $10^{-2}$ or the time limit was over 12 h for the case of homogeneous materials or 24 h for heterogeneous ones. We used an i7–10700K CPU with 8 cores running at 3.79 GHz and with 64 GB RAM. A long time limit was set to analyze the convergence trend of the method for each of the variables. The entire process was divided into two phases. In the first phase, the mechanical characterization was obtained from inverse modeling. After obtaining the final material parameters, the second phase was focused on obtaining the final unpressurized geometry and the stress state. Figure 3 schematizes the full process. In particular, the first phase can be divided into three steps:

(i) At each iteration, the optimization algorithm chose a set of material parameters. Then, the unpressurized geometry was recovered by using the previously described Pull-Back algorithm. For this step, we needed the material parameter of the iteration and the diastolic geometry. In this case, to avoid another iterative process and to reduce the computational cost, we fixed the value of the recovery factor (Latorre et al. 2023). After a previous analysis with different geometries and materials, $k_{zp}$ was set to a value of 0.8, which was an intermediate value for all the analyzed cases.
(ii) As we previously did for the simulated MRI data, we created an FE model with the unpressurized geometry. Then, the systolic state was obtained by adding the systolic load of 120 mmHg and imposing the external tissue support as boundary conditions. Furthermore, we used the measured displacements $u_{\mathrm{{diast}}\rightarrow syst}$ to consider the movements of the surrounding tissues, such as the heart movement. The relative displacements computed in that iteration were recorded ($u_{\mathrm{{diast}}\rightarrow syst}^{iter}$).
(iii) Finally, the error of the cost function value was computed (Eq. 6). If the result was lower than the tolerance, the process moved on to the next step. Otherwise, the algorithm started the loop and evaluated a different set of material parameters.

Once the algorithm provided us with the final GOH parameters, the second phase applied a Pull-Back algorithm without fixing the recovery factor. Therefore, at the end of the process, we obtained not only the material properties but also the unpressurized geometry.

During the development of this methodology, assumptions were made with respect to the wall geometry or the boundary conditions themselves. Therefore, a series of sensitivity analyses were performed to prove the robustness of the methodology.

- When obtaining the unpressurized geometry to create the simulated MRI data, the recovery factor value ranged from [0.6 to 1.2] depending on the material case. However, in the mechanical characterization, we set the value of $k_{zp}$ to 0.8 to avoid another parameter in the optimization. So first, we analyzed how the results varied when considering different values of $k_{zp}$.
- Secondly, the boundary conditions related to the external supports were analyzed. The location of the spine contact could be approximately derived from clinical data, however, the stiffness of the surrounding tissues depends on the individual properties. We therefore studied the robustness of the method by modifying the values of the external stiffnesses of the spine and other tissues.
- Currently, clinical imaging does not allow *in vivo* aortic wall thickness measurement (Bianchini et al. 2023). Therefore, in the theoretical development of the methodology we have considered a constant thickness of 2 mm. However, Smoljkic et al. (2017) reported the importance of the aortic thickness for mechanical characterization. So, we have added a thickness sensitivity study, where we have analyzed how the estimation of mechanical properties was affected by a $\pm 10\%$ and $\pm 20\%$ variation of the thickness. We have simulated MRI data using FE models with 1.6, 1.8, 2.2, and 2.4 mm of wall thickness. All cases were optimized assuming a constant thickness of 2 mm in the iterated model.
- It is possible to non-invasively measure brachial pressure simultaneously with obtaining MRI data but measuring aortic pressure remains a challenge. The difference between the brachial and the aortic pressure may be of the same order of magnitude as the difference between considering 80–120 mmHg and the unknown aortic pressure the patient had during the MRI. Therefore, we assumed the smallest aortic volume as the diastolic phase, with a normal pressure of 80 mmHg and 120 mmHg as systolic pressure (Farzaneh et al. 2019a; Trabelsi et al. 2015). In order to study the effect of assuming a pressure of 80–120 mmHg, a sensitivity analysis of blood pressure was also conducted. We simulated the MRI data with different pressures and increments: 70–110 mmHg, 70–120 mmHg, 80–120 mmHg, 80–130 mmHg, and 90–130 mmHg. For all scenarios, we assumed in the iterative models a pressure range of 80–120 mmHg.

### *In vivo* application and *ex vivo* testing

**Fig. 4:**
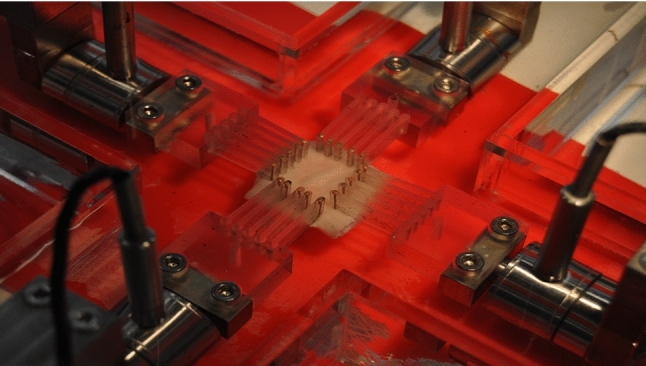
Biaxial test setup used for the *ex vivo* characterization

Alongside the *in silico* models, we also applied the methodology to an *in vivo* case. Preoperative MRI images were obtained from a patient, and with informed consent, a sample of the ascending aorta was collected during the surgical replacement of the ascending aorta. Therefore, it was possible to obtain the mechanical behavior curves both *in vivo* by applying the methodology and *ex vivo* by mechanical testing. We only had one tissue sample from the ascending part of the aorta for experimental characterization. So, we excluded the descending part of the thoracic aortic geometry from the optimization process to ensure the mechanical properties obtained correspond to the sampled area. The *in vivo* mechanical properties were obtained from MRI images. Non-rigid ICP registration was applied to obtain the displacement between the diastolic and systolic phases ($u_{diast\rightarrow syst}^{mri}$). On the other hand, a biaxial test was performed on the sample to obtain its mechanical behavior and compare the results. The biaxial testing machine was operated following the protocol defined by Peña et al. (2018), using a 10 N load cell and force control. Five tests were conducted at different ratios: 1–1, 1$-$0.5, 1–0, 0–1, and 0.5–1, with five cycles per ratio. The reference stress was set at 100 kPa. Based on the dimensions of each sample and the reference stress, the amplitude of the force for each cycle was calculated. The frequency of each cycle was determined by a loading rate of 2 kPa/s. To obtain the behavior curve, we analyzed the last upward segment of the final cycle of the 1–1 ratio. The *ex vivo* test was performed on the sample without differentiating the different layers of the artery to directly compare the results with those obtained in our approach. Figure 4 shows the experimental setup of the biaxial test.

## Results

In this paper, we proposed a methodology to obtain the mechanical response of the material by matching the displacement or strain fields of MRI data with an inverse analysis. The main objective was to adjust the material parameters through a comparison of the relative displacement/strains between systole and diastole. So, we analyzed the error, $J_{0}$, between the real and the simulated variable. However, this value only provided the likeness of the displacement/strain field between diastole and systole. As we worked with *in silico* models and known materials, we can compare the actual material and the resultant of the optimization. Due to the multi-parametric nature of the problem, different combinations of GOH parameters could lead to similar mechanical responses (Liu et al. 2018). Therefore, instead of comparing the differences between real and estimated GOH parameters, we directly compared the stress-stretch curves associated with those GOH parameters. We employed the coefficient of determination ($R^{2}$) between the real behavior curve and the iterated one to quantify their similarity.

### Sensitivity study of GOH parameters

**Fig. 5:**
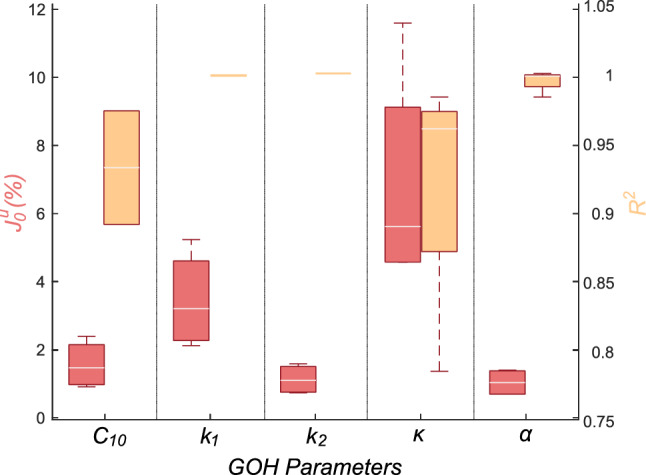
Box-plot of the sensitivity analysis of the different GOH parameters over the displacement cost function ($J_{0}^{u}$) and the $R^{2}$. Red boxes represent the variability of $J_{0}$ for each parameter, and orange boxes the $R^{2}$

Prior to the optimization process, we performed a sensitivity study of the GOH parameters on the displacement field. We selected a set of GOH parameters as the base model, and then we varied ± 10% and ± 20% for each material coefficient. Thus, we analyzed a total of twenty models, evaluating how both $J_{0}^{u}$ and $R^{2}$ varied in each case. Figure 5 shows in red the variability of the displacement cost function obtained by varying each GOH parameter, while orange boxes represent the variability of the $R^{2}$. Material parameters such as $k_{2}$ and $\alpha$, had values close to $J_{0} \approx 0$ and $R^{2} \approx 1$, both with little variability, which indicates that their variation has little impact on the mechanical response. Conversely, $\kappa$ showed a high variability on both variables, indicating a greater relevance in the mechanical response. $\kappa$ and $\alpha$ material parameters are related to each other, as they represent the dispersion and orientation of the fibers, respectively. While small differences in dispersion greatly affected the results, the orientation had hardly any influence.

### Characterization of homogeneous Aortas

**Fig. 6:**
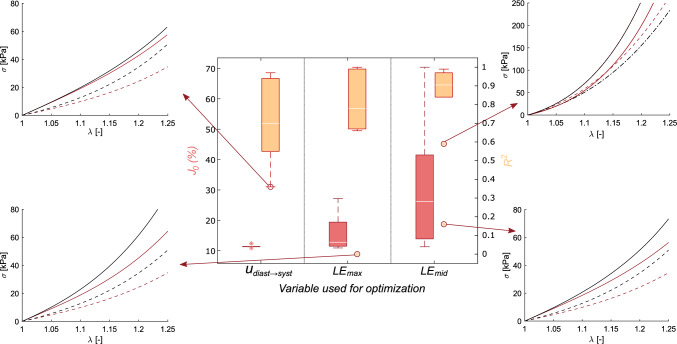
Box-plot of the mechanical characterization process over ten homogeneous cases using three different variables for the optimization process ($u_{diast \rightarrow syst}$, $LE_{max}$ and $LE_{mid}$). Red box plots represent the percentage of error in the cost function, while orange boxes represent the $R^{2}$. Lines represent the mean values, while the asterisk are outliers. There is a representation of the worst $R^{2}$ results for each variable. The stress-stretch curves show in dashed lines the real behavior used in the synthetic models, and in solid lines, the results of the method. The red color corresponds to circumferential behavior and the black to longitudinal behavior

The optimization process was applied to a single geometry, but considering ten ascending aortic materials, both healthy and diseased obtained from the literature. Furthermore, each case was run minimizing three different variables ($u_{diast \rightarrow syst}$, $LE_{max}$, $LE_{mid}$). Figure 6 shows a summary of the results obtained in the 10 cases analyzed for each of the 3 variables used in the optimization. Red box plots represent the cost, $J_{0}$, between the ground truth FE displacements/strains and the resulting displacement/strain field. Orange box plots show the $R^{2}$ between the real and resulting material curves. Additionally, we plotted the stress-stretch curves of the cases with the worst $R^{2}$ for each variable. From this figure, we observed that displacements obtained the smallest cost values, with a median value of $J_{0}=11.4\%$, while maximum and middle principal strain got 12.8% and 26.2%, respectively. In addition to the median value, the interquartile range also increased with the strains. However, when assessing $R^{2}$ the results were completely opposite. In this case, the range decreased with the strains and the median $R^{2}$ value went from 0.69 at the minimization of the displacements, to 0.78 and 0.91 for $LE_{max}$ and $LE_{mid}$.

**Fig. 7:**
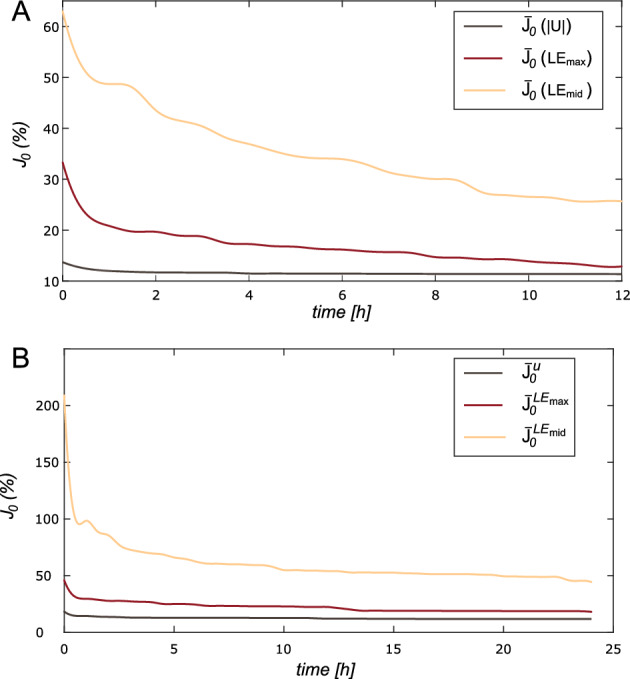
Mean cost error (%) through time (hours). The black solid line stands for displacement minimization, the red line for maximum logarithmic stains, and the orange line for middle logarithmic strains. Results for homogeneous models **A**, and heterogeneous **B**

The pattern-search algorithm needed an initial point to start the evaluations. We used the same starting point for all scenarios. We imposed a time limit of 12 h, achieving a mean number of 286 iterations. However, there were some discrepancies in the convergence of the method depending on the variables used for minimization. Figure 7.(A) shows how the mean cost error decreased in the optimization by using the different variables. We can see again in this figure that the error value is greater in the strains than in the displacement field, especially in the middle logarithmic strain. Although the error values were completely different, the trends of the different variables reached a nearly constant value after a period of optimization. The displacement variable was much faster, with four hours to reach the minima, while the strain took around seven hours.

**Fig. 8:**
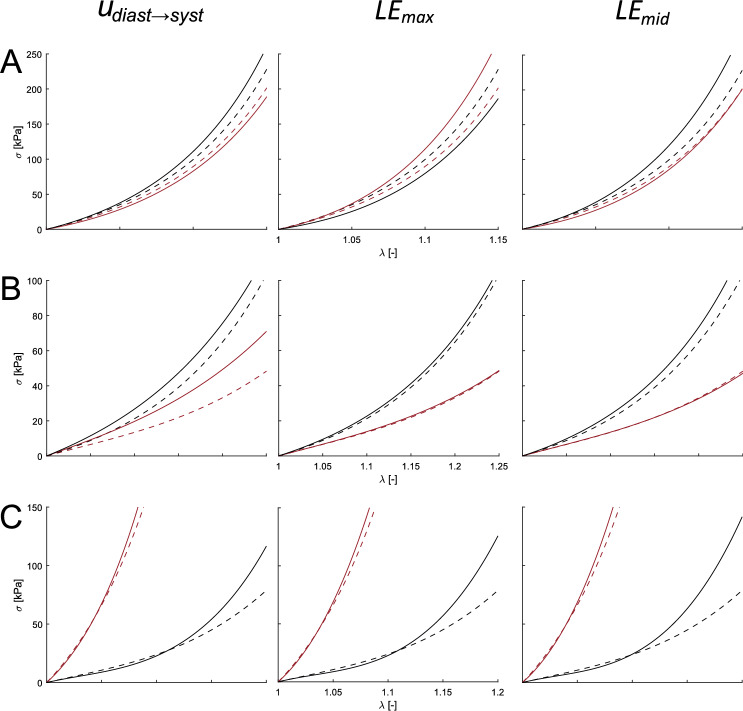
Stress–strain curves of real materials and the resulting ones for cases of isotropic **A**, longitudinal **B**, and circumferential **C** preferential behavior. Red lines represent the circumferential behavior, while black lines the longitudinal. Dotted lines represent the actual *in Silico* curves, and solid ones were the ones resulting from optimizing the displacements ($u_{diast\rightarrow syst}$, left), the maximum logarithmic strains (*LE* *max*, middle), and the median logarithmic strains (*LE* *mid*, right)

There is a wide range of properties of ascending aortas in the literature, there are tissues with predominantly isotropic, longitudinal, or circumferential behavior. Figure 8 shows the curves obtained for different types of material. Rows represent different materials, isotropic, longitudinal, and circumferential behavior. The first column was the results of minimizing displacements, second and third ones of optimizing maximum and middle strains.

**Fig. 9:**
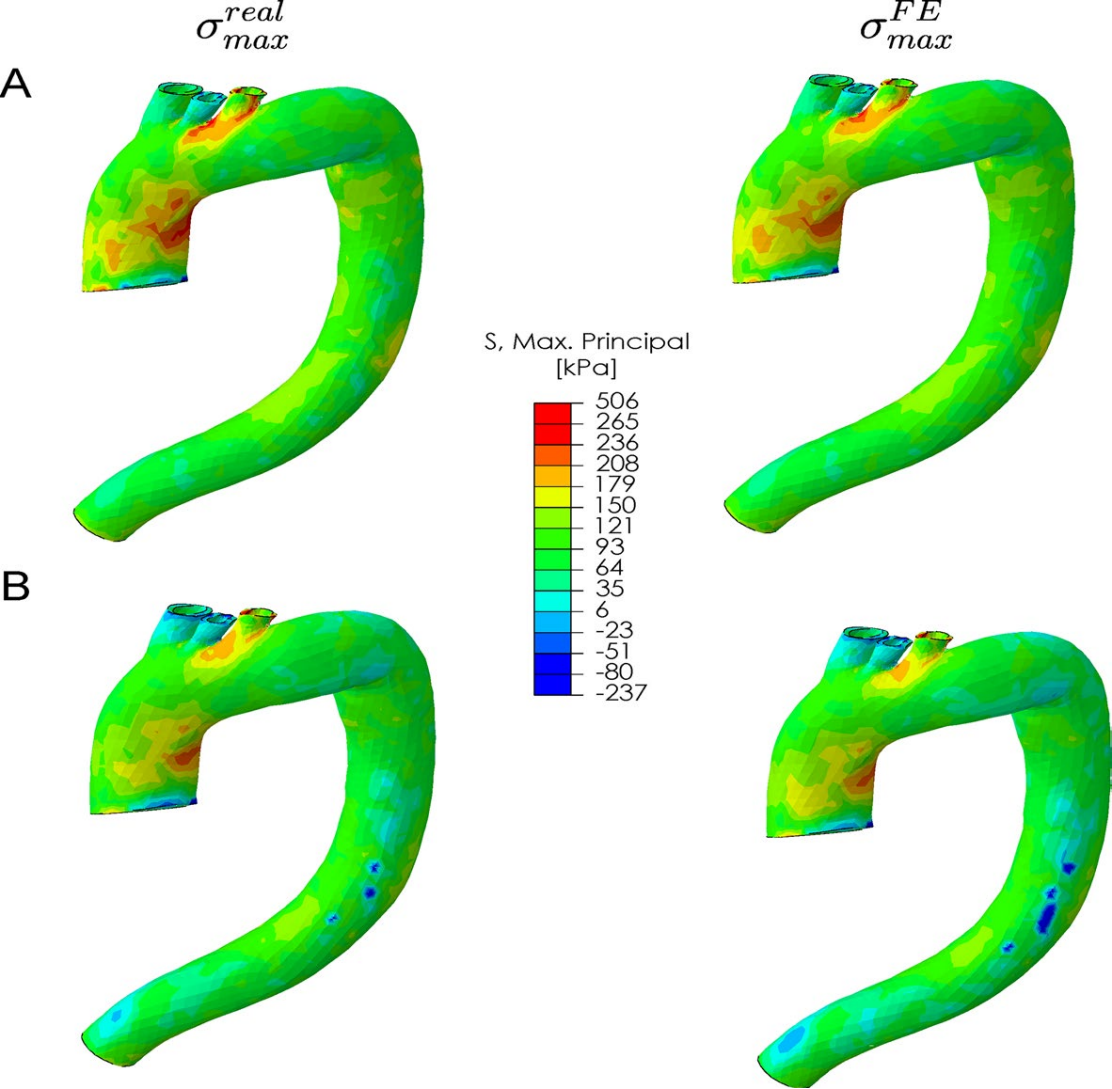
Example of the comparison between the actual maximum principal stress [kPa] map ($\sigma _{max}^{real}$) and the one obtained from our methodology ($\sigma _{max}^{FE}$) in the **A** Homogeneous model and **B** Heterogeneous model

Once the nonlinear properties were obtained, we computed the final ZP geometry and the stress state. Figure 9.(A) shows an example of the actual, $\sigma _{max}^{real}$, and the estimated maximum principal stress, $\sigma _{max}^{FE}$, for a homogeneous model. The stress field obtained was very similar to the real one, both in terms of stress distribution and maximum values.

### Characterization of heterogeneous Aortas

**Fig. 10:**
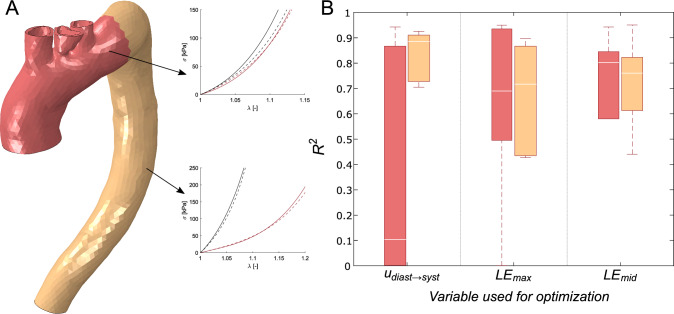
**A** Heterogeneous models with ascending (red part) and descending (orange part) divisions. It shows the real and the resulting behavior curves of the ascending and descending parts, where the dashed lines represent the actual behavior and the solid lines the resulting materials. Red color for circumferential and black for longitudinal. The real material properties correspond to the numbers 3 and 13 of table 1. **B** Box plot of $R^{2}$ for the ascending aorta in red and descending in orange

In this section, we analyzed a heterogeneous aorta by considering different material properties for the ascending and descending parts. The descending portion was considered healthy, while the ascending one was analyzed both as healthy and with aneurysm properties. We analyzed a total of five heterogeneous models. In this case, the optimization time limit was set to 24 h. However, the cost values remained nearly constant from eleven hours for displacements, and thirteen hours for logarithmic strains, as seen in Fig. 7.(B). Figure 10.(A) shows an example of a heterogeneous model with the results for both regions. Figure 10.(B) represents the box plots of the resulting $R^{2}$ for ascending, and descending parts for the three variables used during the optimization. After analyzing the five ascending regions, the mean $R^{2}$ values were 0.1, 0.7, and 0.8 by using the displacements and the maximum or middle logarithmic stains respectively. In the descending aorta, the results were 0.9, 0.72, and 0.8. Moreover, the stress distribution obtained in these models was almost the same as that observed in the real models. Figure 9.(B) shows an example, where the similarity of both stress maps can be appreciated. As in the previous case, the stress field obtained was very similar to the one considered real.

### Sensitivity analysis

**Fig. 11:**
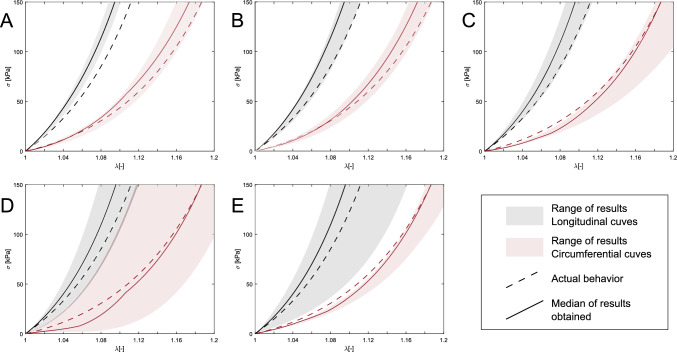
Results of the stress-stretch curves for the different sensitivity analyses minimizing the variable $LE_{mid}$. The base model had the material properties number seven of Table 1. (A) Results of varying the recovery factor between 0.6 and 1.2. (B) Results of changing the stiffness of the external tissues between $\pm 10\%$ and $\pm 50\%$ respect the base model. (C) Results of the lumen pressure sensitivity analysis. (D) and (E) show the results of analyzing different wall thicknesses using $LE_{mid}$ and displacements in the minimization respectively. The plots show the whole range of results for each analysis. Dashed lines represent the mechanical behavior of the *in silico* model, and the solid lines are the results of the process. The red color is the circumferential behavior, and the black color is the longitudinal behavior

In this section, we analyzed how the results varied for the different sensitivity analyses performed. First, the recovery factor value was set at 0.6, 0.7, 0.8, 0.9, and 1. Figure 11.(A) shows the variation of the resulting curves by considering the different values for a homogeneous model and minimizing the $LE_{mid}$. Results suggested that the characterization obtained created an overestimation of tissue stiffness in both directions. However, both the trend and the values obtained were very close to the actual behavior.

As a first approximation to validate the methodology, we considered that the external stiffness of the tissue was equal both when simulating the MRI data and in the iterated models. The area of contact with the spine can be approximately inferred from medical imaging. In addition, the stiffness value of each zone had been obtained from average values (Moireau et al. 2012; Pagoulatou et al. 2021). Therefore, we analyzed how it can affect the variation of the external tissue stiffness. We studied the mechanical variation of modifying the stiffness of the surrounding tissues by $\pm 10\%, \pm 20\%, \pm 30\%, \pm 40\%$, and $\pm 50\%$. Figure 11.(B) shows the variation of the curves. As in the previous case, there was an overestimation of stiffness, but in all cases, the results were similar to the real ones.

Since no measurements of the diastolic and systolic pressures were taken during the acquisition of MRI images, we assumed 80 mmHg of pressure for the diastolic geometry and 120 mmHg for the systolic state in our optimization methodology. Figure 11.(C) shows the stress-stretch range after considering ranges of diastolic-systolic pressures of 70–110, 70–120, 80–120, 80–130, and 90–130 mmHg minimizing the $LE_{mid}$ variable. Both the trends of all results and the averages were very similar to the actual behavior of the tissue.

We simulated MRI data using FE models with 1.6, 1.8, 2.2, and 2.4 mm wall thickness. Figure 11.(D) and (E) shows the results obtained in the analysis by minimizing $LE_{mid}$ and displacement fields, respectively. It is important to note that the figure shows the full range of results obtained. Interestingly, it is worth noting that when optimizing $LE_{mid}$ the circumferential curves corresponding to the $\pm 20\%$ thickness models obtained curves quite far from the actual behavior, failing to describe the mechanical behavior. However, when using the displacement field in the minimization, only the longitudinal curve of the 1.6 mm thick model failed.

### *In vivo* application and *ex vivo* testing

**Fig. 12:**
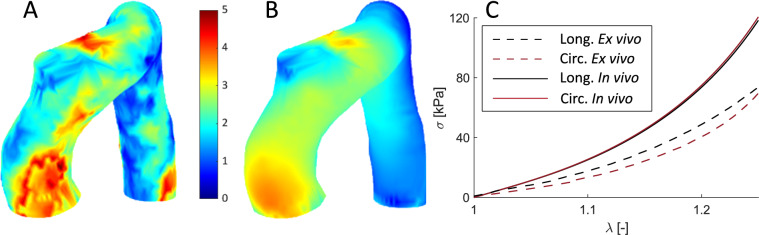
**A** Displacement field [mm] obtained from non-rigid ICP registration from MRI data. **B** Displacements [mm] obtained from the methodology. **C** Stress-stretch biaxial curves of the *in vivo* and *ex vivo* testing, in solid and dashed lines, respectively. The red line for circumferential behavior and the black for longitudinal

Finally, we compared the *in vivo* and *ex vivo* mechanical responses of a patient who underwent surgery. Therefore, we directly applied the optimization methodology to real MRI data. In this case, due to the low resolution of the sagittal cine, the strain fields presented a lot of noise and unrealistic distributions, so, the methodology was only applied by minimizing the displacements variable. Moreover, the previous sensitivity analysis showed that the displacement variable obtained better results when the wall thickness was not known. Figure 12.(A) shows the displacement field over the lumen surface between diastolic and systolic phases registered by non-rigid ICP from MRI data. Figure 12.(B) presents the relative displacement obtained in the methodology. Although the *in vivo* displacement field presented a more irregular distribution compared with the computed one, the results showed a similar distribution, where areas of maximum and minimum displacements in the *in vivo* and in *silico* examples are mostly overlapping, especially in the ascending part. From the biaxial test, it was possible to obtain the GOH parameters and then the stress-stretch curve. Figure 12.(C) shows the curve of the sample in a dashed line. It can be observed that in both directions, longitudinal and circumferential, the tissue behaves in the same way, showing an isotropic behavior. A very similar behavior was observed with the *in vivo* methodology, where the longitudinal direction is represented by a black continuous line and the circumferential direction by a red continuous line.

## Discussion

We presented a methodology to obtain the nonlinear anisotropic properties of the ascending aorta from MRI data. The process was based on the ability to segment the geometry of the aorta and to measure the relative displacement or strain field between diastole and systole from MRI data. We implemented an inverse finite element process, where we optimized the GOH material parameters by minimizing the difference between a variable measured from clinical data and the computational one. We considered three different variables to minimize, the modulus of the displacements and maximum and middle principal logarithmic strains. Previous to the optimization process, the influence of the different GOH parameters was analyzed to adapt the pattern-search algorithm. Following the sensitivity analysis results, we imposed greater weight in the process on parameters such as $\kappa$ and $k_{1}$. Although the fiber orientation, $\alpha$, showed very little influence on the mechanical behavior when varying its value, it did have a great influence on whether the behavior is more isotropic, more longitudinal, or more circumferential. Therefore, it was also given greater weight in the optimization.

First, we analyzed the homogeneous models, where the whole aorta was analyzed with a single material. In the literature, it is common to find methodologies to obtain the mechanical properties of tissues, but most of them used only a few cases for numerical validation (Liu et al. 2018; Zhang 2021), or directly applied the methodology on *in vivo* data and compared it with *ex vivo* experiments (Liu et al. 2019). In this work, we validated the methodology with different sets of *in silico* models with diverse materials and boundary conditions before applying it directly to real data. In many clinical applications, time is normally an important factor, and this method set the maximum optimization time at twelve hours. However, the cost function error decreased quickly in the first two hours, and then it became almost constant from 4 h for displacement minimization and 6–8 h for strain optimizations. This meant the convergence time depended on the variable of minimization, but in all cases, they required less time than similar inverse approaches, where the time required was 1–2 days (Liu et al. 2017) or even weeks (Wittek et al. 2016). Apart from the time, the major difference between using displacements or logarithmic strains in the optimization process was the cost value ($J_{0}$). In comparison with the strains, displacements always obtained a cost value really small, mainly because of two reasons. Firstly, we imposed the measured displacements as boundary conditions in some nodes to mimic the physiological displacements, so in those nodes, the cost will always be zero. Secondly, principal logarithmic strains, especially middle ones, had values close to zero. So, using the cost function 6 in areas with low strains field with values close to zero, the value of the error soars. Conversely, the coefficient of determination was higher for strain minimization rather than for displacements. In fact, $LE_{mid}$ had the higher cost values but the best $R^{2}$ results, with the higher median value and the less variability. In reality, the value of the cost function is not as significant as the fact that it decreases during minimization. For instance, some studies analyzed the decrease of the node-to-surface distance to verify the solution rather than giving importance to the specific value (Liu et al. 2018, 2019). While the cost value measured the similarity between the target and the iterated variable, the $R^{2}$ showed the proximity of the iterated GOH parameters with the actual mechanical behavior. The trend of achieving the best $R^{2}$ performances with the variables that had the bigger cost error could be correlated with the pattern-search algorithm. We hypothesized that having such huge values in strain cost functions could avoid getting stuck in local minima. Nevertheless, with all the variables used in the minimization, the results suggested a good match between the real and the resulting behavior. Even those cases with really low $R^{2}$ values got similar curves, as shown in Fig. 6. In some cases, we obtain $R^{2}$ lower than 0.5 and yet we had very similar curves. This was because the stiffer materials have almost vertical behavior curves, which causes the value of $R^{2}$ to drop drastically.

Normally, the studies focused on determining the ascending or descending thoracic aorta properties, by analyzing a small homogeneous portion of the aorta or considering the whole geometry as homogeneous (Liu et al. 2017, 2018, 2019; Zhang 2021). Farzaneh et al. (2019a) developed a methodology to recover local stiffness in ATAA, providing the importance of not considering homogeneous tissues. Assuming isotropic behavior, they were able to obtain stiffness maps. However, they validated the methodology by comparing only the stiffness distribution instead of the stress/strain response. In this study, we compared the mechanical response of ascending and descending aorta materials. Although we only considered two parts, it could be applied to more regions, which would increase the computational cost. Heterogeneous models provided similar results, however, as the number of optimized parameters moved from five to ten, the time until convergence changed. In these models, the average time from which it stabilized the cost function was 11 h for displacements and 13 for strain comparisons. Instead of optimizing ascending or descending material parameters together, we could have minimized each region independently. This approach would have reduced computational cost; however, it produced discontinuities at the junction between the ascending and descending regions. Additionally, unrealistic stress concentrations appeared in that area. The heterogeneous results showed the same trend with $R^{2}$ and $J_{0}$ as homogeneous models. The logarithmic strains showed similar fitting results for both the ascending and descending parts. However, the displacements fitted the descending part better but gave poor results for the ascending aorta. Overall, the methodology successfully characterized the mechanical behavior for isotropic, predominant longitudinal, or circumferential materials. As regards the variable to be optimized, the relative displacement field provided the best fit in terms of cost function values and its convergence time was shorter. Displacement optimization was already employed in different approaches (Akyildiz et al. 2016; Torun et al. 2022), however, this variable considers the motion as a rigid solid. Therefore, some approaches used the stress field as an optimization variable (Liu et al. 2017; Zhang 2021), but strain fields performed better results and neglected the motion of the aorta (Zhang 2021). Although it took more time to converge, principal logarithmic strains provided a mechanical response closer to the actual performance as shown in Fig. 8. Therefore, the cost function values were not reliable information on the quality of the adjusted results.

In both homogeneous and heterogeneous models, the stress field obtained was very similar to the real distribution. Regions with high-stress levels have been related to areas of rupture or dissection of the aorta (Trabelsi et al. 2015; He et al. 2021; O’Rourke et al. 2022), so this information could be very useful in the clinic. The correct determination of the stress field and its gradient across the aortic wall thickness will depend on the quality of the mesh. During the optimization process, we only compared the displacements and strains on the lumen surface. To minimize computational cost, we used coarse meshes with just one element across the wall thickness, which led to a mean stress distribution. To enhance spatial resolution, we could re-mesh the geometry with finer elements, utilizing the unpressurized geometry and nonlinear properties provided by the methodology. However, since the actual wall thickness of real patients is not currently available from clinical images, the resulting stress distribution across the thickness will remain approximate. The degree of similarity when applied to real data will also depend on the heterogeneity of the tissue and the variation in thickness (Raut et al. 2013; Cavinato et al. 2019).

Considering a Pull-Back algorithm was a key point in our methodology to obtain the nonlinear material properties. However, the unpressurized geometry was conducted in the material optimization by fixing the value of $k_{zp}$ to 0.8. We additionally analyzed the influence of varying this variable from 0.6 to 1. Figure 11.(A) shows that there was no relevance of this parameter for obtaining the mechanical properties. The value of the recovery factor affected the unpressurized geometry and consequently had an impact on the stress distribution. Therefore, after obtaining the mechanical properties, we performed another optimization process to obtain the real value of $k_{zp}$, and thus, the zero pressure geometry. Unlike most studies, we incorporated the external tissue support as a boundary condition since neglecting surrounding tissues created an unrealistic motion pattern (Pagoulatou et al. 2021; Moireau et al. 2012). The location of the spine could be inferred from the clinical image. However, it is impossible to obtain the stiffness of the tissues *in vivo*. We used the same stiffness values for simulating the MRI data and for the optimization process. It was tested to modify the stiffness in the optimization process by $\pm 10--50\%$ concerning the stiffness used to generate the simulated MRI data. The results showed similar behavioral curves for the analyzed values, suggesting that for high levels of stiffness, it was not of great relevance. The blood pressure sensitivity study shows a relatively minor impact when changing diastolic and systolic blood pressure over realistic ranges. When analyzing the median stress-stretch curves for wall thickness variation, Fig. 11.(D) and (E) shows that the results closely align with the actual behavior. On the other hand, in specific cases, particularly when minimizing the $LE_{mid}$ variable, assuming a constant thickness of 2 mm may impact the accuracy of the results. When using the displacement field in the minimization, only the longitudinal curve of the 1.6 mm wall thickness was far from the actual one. But for the other cases, the displacement variable achieved better fits. Therefore, when the thickness is very different from the estimated thickness, in this case 2 mm, the displacement variable provided results with trends more similar to the real behavior. This means that the strain variables were more sensitive than the displacement variable to thickness variation. Although there was greater variation in the behavioral curve when different aortic thicknesses were considered, the median of the results of minimizing $LE_{mid}$ and displacement closely matches the actual behavior. This suggests that the variation was primarily due to specific cases, such as a thickness change of 20%, while in cases with a 10% variation, the methodology effectively captured the mechanical properties.

We have been able to apply the methodology to an *in vivo* case. It has only been applied using the displacement field as the variable to be minimized due to the heterogeneous spatial resolution (through-plane spacing is about four times in-plane spatial resolution). This heterogeneous spatial resolution caused discontinuities to appear when deriving the strain field from the displacement data. Although the strain fields provided better results at a theoretical level, the displacements allowed for a much shorter convergence time, which would facilitate their use in clinical diagnostics. In addition, we have seen in the thickness sensitivity analysis, that when the thickness is not known, which is very likely the case considering the limitations of current imaging techniques (Bianchini et al. 2023), the optimization of the displacement field obtains better results. Higher-resolution sagittal cine images or different methods of motion quantification may allow applying the methodology to strain fields. After applying the methodology, we were able to obtain an estimate of the displacement field in the ascending zone of the aorta. However, the estimate for the descending part was less accurate due to the fact that the sample analyzed corresponds to the ascending region, which made it less representative of the behavior of the material in that zone. The stress-stretch curves obtained *in vivo* and *ex vivo* showed a similar trend, with highly isotropic behavior. Moreover, the tissue presented in both approaches had a softer mechanical response compared to those obtained in the literature. However, the *in vivo* curves were stiffer than those obtained in the biaxial test. This discrepancy may be due to different reasons. For instance, only a portion of the ascending aorta was characterized *ex vivo* which may induce a slightly different mechanical response from the *in vivo* (Trabelsi et al. 2015).

### Limitations

The proposed methodology successfully characterizes different nonlinear, anisotropic materials for homogeneous and heterogeneous models. Nonetheless, this study has some limitations that have to be mentioned. While different material properties were analyzed, both healthy and diseased, the same geometry was considered in all cases. Although we have studied the influence of thickness, we used a uniform wall thickness, while the thickness itself is a major contribution of the biomechanical behavior (Raut et al. 2013; Smoljkic et al. 2017; Wang et al. 2023). A non-uniform thickness could affect the peak wall stress by 20%, but considering nonlinear properties has a greater impact than realistic thickness (Raut et al. 2013). Moreover, Cosentino et al. (2023) demonstrated that knowing the local thickness will not be decisive for the mechanical stiffness variations. The optimization time was high and depended on the variable used during the minimization. Traditionally, inverse modeling methods involved high computational costs. Other approaches tried to fit GOH material parameters from clinical imaging, however, variables such as pressure or lumen diameter were not enough for a proper characterization (Smoljkic et al. 2017). Liu et al. (2019) used an inverse model based on a multi-resolution direct search that provided material properties for *in vivo* ATAA in around 2 h. However, this type of algorithm was only valid for homogeneous materials. Newer methodologies, such as Bayesian optimizations could reduce computation costs in the optimization of different tissues and have already provided good results for atherosclerotic plaques (Torun and Swaminathan 2019; Torun et al. 2022). As we have seen, the coefficient of determination was not a reliable measure to determine the fit of materials with such exponential behavior. Especially for stiff tissues, since it offered very low fitting values but the curves had a similar behavior. Therefore, another coefficient should be used for future error quantification to better measure the goodness of fit. This work was primarily a theoretical framework, so we only tested the methodology with a single *in vivo-ex vivo* case for validation. Therefore, in future work, it would be necessary to test the methodology with more *in vivo* cases.

In conclusion, despite its above-mentioned limitations, the presented inverse modeling methodology offers a means to estimate the nonlinear anisotropic properties of the aorta. Moreover, the methodology attempts to replicate physiological conditions of the aorta, such as external tissue contact and heart motion. *In silico* validation of the methodology has demonstrated its accuracy and robustness. This study has been mainly theoretical and has only been validated with one *in vivo* and *ex vivo* case. Therefore, it should be tested with more patients.
